# Role of Nutritional Supplements in Sport, Exercise and Health

**DOI:** 10.3390/nu15204429

**Published:** 2023-10-19

**Authors:** Andreina Alfieri, Stefania D’Angelo, Filomena Mazzeo

**Affiliations:** 1 Department of Movement Sciences and Wellbeing, Parthenope University, 80133 Naples, Italy; andreina.alfieri@uniparthenope.it (A.A.); filomena.mazzeo@uniparthenope.it (F.M.); 2 Department of Economics, Law, Cybersecurity and Sports Sciences, Parthenope University, 80133 Naples, Italy

Health promotion requires good nutrition and an adequate lifestyle, which together contribute to people’s well-being. In recent years, an increasing number of scientific research has demonstrated how following a correct diet and eating healthy foods can reduce non-communicable diseases, such as heart syndromes, cancer, and diabetes.

In recent years, nutritionists have increasingly considered dietary protocols that provide greater importance to the interactions and synergies between various foods rather than evaluating the beneficial effects of a single food. Many studies have evaluated the correlation between dietary patterns and human well-being and proposed how specific combinations of foods have clearer beneficial effects than individual foods.

Therefore, following a high-quality diet rich in a variety of foods, rather than a nutritional model rich in a single food such as cereals, is becoming a suitable dietary strategy to prevent many syndromes. It has been shown that diets such as the Mediterranean diet, the Dietary Approaches to Stop Hypertension (DASH) diet, and the Mediterranean-DASH Intervention for Neurodegenerative Delay diet are associated with less cognitive decline and a lower risk of Alzheimer’s disease [1]. These nutritional protocols highlight the need for balanced consumption of a variety of foods, particularly vegetables, legumes, legumes, poultry, and fish.

The Mediterranean diet is a nutritional protocol characterized by foods rich in phytochemicals, such as polyphenols, which includes a large intake of plant foods, such as fruit, vegetables, dried fruit, and olive oil; instead, it involves limited consumption of red meat and processed meat, butter, alcohol (red wine), and sugary drinks [2]. Over the years, many scientific works have highlighted the healthy action of the Mediterranean diet in decreasing the incidence of many diseases, including cancer, metabolic syndrome, and neural degeneration. Instead, adverse effects, such as an increased risk of hypertension, glucose intolerance, metabolic syndrome, abdominal obesity, and hyperlipidemia, have been noted in subjects following diets characterized by foods rich in fats, sugars, and proteins. of animal origin [1].

The importance of diet and the benefits of taking, when necessary, food supplements for health and the prevention of numerous syndromes have been documented in numerous scientific studies; numerous scientific data demonstrate the existence of a correlation between the intake of specific nutrients or food supplements and the prevention of chronic syndromes such as cancer, osteoporosis, and heart disease; numerous clinical research have demonstrated how numerous chronic diseases can be prevented simply by a healthy diet, for example, a diet with a low content of saturated fats and cholesterol, a limited amount of sodium, and, on the contrary, with a high percentage of foods of plant origin [3].

Most micronutrients are included in the European Union Register on Nutrition and Health Claims and play a role in the immune system functioning, particularly in immune response [4]. A food supplement refers to a preparation intended to supply a nutrient that is missing from a diet. It is a product taken orally generally, has one or more ingredients intended to supplement one’s diet, and is not considered food. It can be either an extracted substance from food sources or a synthetic compound and can contain one or more of the following dietary ingredients: vitamins, minerals, herbs or other botanicals, and amino acids. So, the main categories of dietary supplements are vitamins, dietary minerals, proteins and amino acids, essential fatty acids, probiotics, and natural products [5].

In some conditions, the controlled intake of micronutrients could be fundamental for non-communicable diseases, such as cancer, diabetes mellitus, cardiovascular diseases, and respiratory diseases, which are considered the main causes of death globally. Observational studies have often reported an inverse association between a high intake of fruit, vegetables, fish, and the risk of disease; these data suggest how specific vitamins and minerals could handle these beneficial effects [2].

Improper nutrition and micronutrient deficiencies negatively affect health. Although improving the quality of food intake is essential to address these problems, food supplements and/or food fortification could help meet the needs of those at risk of deficiencies. Food supplements are widely used and can improve health if properly taken. Scientific data show how some supplements can improve health via different actions. The most popular dietary supplements are multivitamins, calcium, and vitamins B, C, and D. For example, calcium supports bone health and vitamin D helps calcium absorption.

Until a few years ago, scientific studies on food supplements were not widespread; therefore, knowledge about their effects was limited. The use of supplements has increased significantly over the last 30 years, and they are still the subject of great consumer interest [6,7]. These products are available in many forms (capsules, tablets, soft gels, gelatin capsules, liquids, powders, and chewable preparations); they are sold as individual products or as combinations of mineral salts, vitamins, herbs, amino acids, fatty acids, and other dietary components.

The intake of food supplements of essential mineral salts and vitamins is essential when nutritional needs are not met through the diet. Instead, the role of nutritional supplementation, when nutritional sufficiency has already been achieved through the diet, is still strongly debated; in fact, possible harmful effects have been proved in the case of some micronutrients if taken in quantities exceeding the recommended dosage.

While a series of nutritional products on the market supply nutritional support to subjects suffering from chronic diseases, investigations of the effectiveness of a vast number of nutraceuticals aimed at improving athletic performance are also exploding in the scientific literature.

The use of sports supplements to improve performance is very common in athletes. Extracts from edible plants may prevent stress-associated cell damage, reactive oxygen species generation, and physiological processes, such as metabolism and inflammation [8]. Nutrition, therefore, plays an essential role in the daily life of a player, and this is positively reflected in psychological well-being and, above all, in sports performance [9].

Well-designed nutritional approaches can improve sporting performance and recovery from sporting activities. Appropriate carbohydrate intake has been shown to achieve better endurance, whereas the co-ingestion of protein and carbohydrate after exercise can result in better recovery [10]. Nutritional ergogenic aids are substances that can give athletes an advantage for competition purposes, generally acting to improve energy metabolism and body composition. It has been shown that ingestion of beta-alanine can increase muscle carnosine levels in skeletal muscle.

Caffeine is a central nervous system stimulant of the methylxanthine class; it is extracted from some plants and is commonly ingested in the form of coffee, tea, cola drinks, and energy drinks. Creatine is derived from the amino acids methionine, glycine, and arginine. It is found primarily in skeletal muscles and the brain. Most people obtain creatine from seafood and red meat at levels much lower than those found in synthetically produced creatine supplements. Caffeine and creatine are ergogenic aids that can improve the performance of athletes during competitions. Vitamins and mineral salts are fundamental in energy metabolism and hemoglobin synthesis; they promote bone health and immune function and act as antioxidants that protect the body from oxidative damage [10]. Since physical activity increases energy expenditure, exercise can increase the turnover of B vitamins. An increase in the rate of sweating caused by regular physical activity can also increase the loss of mineral salts such as zinc and magnesium and zinc [3]. As a result, athletes may need increased micronutrient intake, especially those who train for long periods or play high-intensity sports with significant energy expenditure.

Nutritional status is a major factor that affects the performance, endurance, and general health of athletes and individuals interested in overall wellness. Most athletes use various nutritional and dietary supplements. Many studies highlight how an adequate diet before, during, and after training, as well as a match, can improve performance. Moreover, the evolution of lifestyles, proteins, vegetarian and vegan diets, a culture of additives, and the pressure on sports stakeholders have led to a significant increase in the consumption of supplements. In any case, healthy nutrition does not need to be integrated, except for training conditions, and the incorrect use and abuse of the substances are serious health and sports lawfulness problems. Therefore, the right nutritional guidelines are an essential part of the best training programs to aid in adaptations, peak sports performance, and injury prevention. Nutrition, therefore, plays a fundamental role in the daily life of not only athletes; this role is certainly reflected in psychological well-being and, above all, in sports performance.

Based on the recent understanding of the needs of sportspersons, nutrition has the potential to improve performance. Sports nutrition professionals should work closely with athletes and coaches to ensure that players consume the correct quantities and types of food for training and race.

Supplements are not subject to legislation that concerns drugs, but manufacturers must exclusively guarantee that they are safe for health, as is the case with food. Plant extracts used in supplements are more complex than simple minerals or vitamins because they contain a mixture of substances, some of which may be beneficial to our health. When these effects are proven by scientific studies, EFSA (European Food Safety) expresses a positive opinion on specific claims. The claims relating to health effects authorized by EFSA are not very many, but in any case, all supplements have indications for use and recommended doses based on scientific knowledge on the subject.

The studies presented in this Special Issue describe new scientific knowledge that is essential and fundamental in further understanding the role of nutritional support. As the biological mechanisms underlying noncommunicable syndromes continue to be elucidated and the causes and consequences of diet-related conditions are better characterized, new intervention plans may be realized, studied, and evaluated. These results will be fundamental for drafting and developing new evidence-based dietary guidelines and could help professionals teach their patients or clients to adopt healthy eating behaviors. However, a well-balanced diet is still the recommended approach to ensure an adequate intake of essential macronutrients and micronutrients. Naturally, there are cases in which supplementation plays a fundamental role in the treatment of vitamin and mineral salt deficiencies. However, supplementation for subjects who already take sufficient doses through the diet is still debated; its effectiveness and necessity are greatly questioned, and potential negative effects are currently still under investigation.
